# Gestational diabetes mellitus, hypertension, and dyslipidemia as the risk factors of preeclampsia

**DOI:** 10.1038/s41598-024-56790-z

**Published:** 2024-03-14

**Authors:** Farah Aziz, Mohammad Fareed Khan, Amna Moiz

**Affiliations:** 1 Department of Basic Medical Science, College of Applied Medical Science, King Khalid University, Abha, Saudi Arabia; 2 Department of Infection Prevention and Control, The Specialist Hospital, Abha, Saudi Arabia; 3 Medical City, King Khalid University, Abha, Saudi Arabia

**Keywords:** Gestational diabetes mellitus, Preeclampsia, Pregnancy, Hypertension, Dyslipidemia, Biochemistry, Biomarkers, Endocrinology

## Abstract

Gestational diabetes mellitus (GDM) is a known risk factor for gestational hypertension which further progress toward conditions like proteinuria, dyslipidemia, thrombocytopenia, pulmonary edema leading to Preeclampsia (PE). Pregnancy can be a challenging time for many women, especially those diagnosed with GDM and PE. Thus, the current prospective study investigates the association of OGTT glucose levels with systolic and diastolic blood pressure and lipid profile parameters in pregnant women diagnosed with GDM and PE. A total of 140 pregnant women were stratified into GDM (n = 50), PE (n = 40) and controls (n = 50). Two hour 75 g oral glucose tolerance test (OGTT) was performed for screening GDM. Biochemical parameters analysis of OGTT, total cholesterol (TC), triglyceride (Tg), high density lipoprotein-cholesterol (HDL-C), low density lipoprotein-cholesterol (LDL-C), urinary albumin and creatinine were tested to find urinary albumin creatinine ratio (uACR). Statistical analysis was performed using ANOVA followed by post hoc test and regression analysis. Among the studied groups, GDM and PE groups showed no significant difference in age and increased BMI. Increased 2 h OGTT & TC in GDM group; elevated uACR, systolic/diastolic blood pressure, Tg, HDL-C, LDL-C in PE group was observed and differ significantly (*p* < 0.0001) with other groups. A significant positive effect of 2 h OGTT was observed on blood pressure (R^2^: GDM = 0.85, PE = 0.71) and lipid profile determinants (R^2^: GDM = 0.85, PE = 0.33) at *p* < 0.0001. The current study concludes that glucose intolerance during the later weeks of pregnancy is associated with gestational hypertension and hyperlipidemia as a risk factor for PE. Further research is needed for a detailed assessment of maternal glucose metabolism at various pregnancy stages, including the use of more sensitive markers such as C-peptide and their relation to pregnancy-related hypertensive disorders.

## Background

Gestational diabetes mellitus (GDM) and preeclampsia (PE) are common pregnancy-related problems responsible for maternal and fetal mortality and morbidity^1^. GDM is characterized as the initial diagnosis of glucose intolerance during pregnancy^2^. The International Association of Diabetes and Pregnancy Study Groups (IADPSG) has formed the diagnostic criteria for GDM based on the findings of the Hyperglycemia and Adverse Pregnancy Outcome (HAPO) study^3^. The pathophysiological mechanisms in both GDM and PE entail oxidative stress, pro-inflammatory factor release, and vascular endothelial dysfunction, which conjointly raise the risk of future maternal diabetes and cardiovascular disease, suggesting a link between GDM and PE^4,5^.

According to the recent global report by the International Diabetes Federation (IDF), GDM complicates up to 80.3% of all pregnancies^6^. GDM is a major risk factor for gestational hypertension, which causes approximately 30,000 deaths per year among women^7^. Hyperglycemia induces a proinflammatory milieu and cytokine imbalance, resulting in placental vascular alterations, whilst insulin directly causes placental inflammation. One or more of the following conditions, such as proteinuria, dyslipidemia, thrombocytopenia, pulmonary edema, or persistent neurological symptoms, are also additional diagnostic indicators of gestational hypertension and PE^8^. The progressive PE manifests renal impairment, hepatic impairment, hemorrhagic stroke, ischemic acute respiratory distress syndrome, placental abruption, preterm labor, and delivery. A study conducted in Saudi Arabia by Subki et al. revealed that hypertensive disorders during pregnancy had a prevalence of 2.4%, with PE being the most common subtype at 54.9%^9^. It has been proposed that women with GDM exhibit a greater prevalence of dyslipidemia than their normoglycemic counterparts^10^. Additionally, a recent finding addresses the pathophysiology of PE, including dysregulations of fetal-maternal lipid metabolism^11^.

Nonetheless, an altered lipid profile causes vasoconstriction and endothelial dysfunction by suppressing endothelial prostacyclin and nitric oxide (NO), increasing oxidative stress. Although physiological hyperlipidemia is non-atherogenic until allied with associated severe hypertension^12^. The Fifth International Workshop-Conference on GDM recommended the treatment of GDM with lifestyle interventions like medical nutrition therapy, physical activity, weight management, and continuous glucose monitoring^13^. Pharmacological therapies like sulfonylureas and biguanides are contraindicated in GDM as they readily cross the placenta and are found to be associated with a higher rate of neonatal hypoglycemia, macrosomia, and increased neonatal abdominal circumference, hyperbilirubinemia^14,15^. Insulin is the recommended medication for treating hyperglycemia in GDM, and a low-dose aspirin at 12 to 16 weeks of gestation to lower the risk of preeclampsia^13^.

With the hypothesis that elevated maternal glucose concentrations during pregnancy can affect blood pressure and lipid profiles, consequently leading to PE, the present prospective study examined the association of OGTT glucose with systolic, diastolic blood pressure, and lipid profile parameters in pregnant women diagnosed with GDM and PE. This study is regionally important and has not been conducted before in Abha, the southern region of Saudi Arabia. However, a decade earlier, PE and eclampsia partake was observed on maternal and fetal mortality.

## Materials and methods

### Study setting

The study was conducted in the Basic Medical Sciences department of King Khalid University in collaboration with AlKhamis Maternity and Children Hospital, Khamis Mushayt. The study was approved by the ethical committee of King Khalid University, Abha, Saudi Arabia under approval number ECM#2021-601. An informed consent was obtained from the study subjects and methods were performed in accordance with the relevant guidelines and regulations of Helsinki.

### Study participants inclusion and exclusion criteria

**Figure 1:**
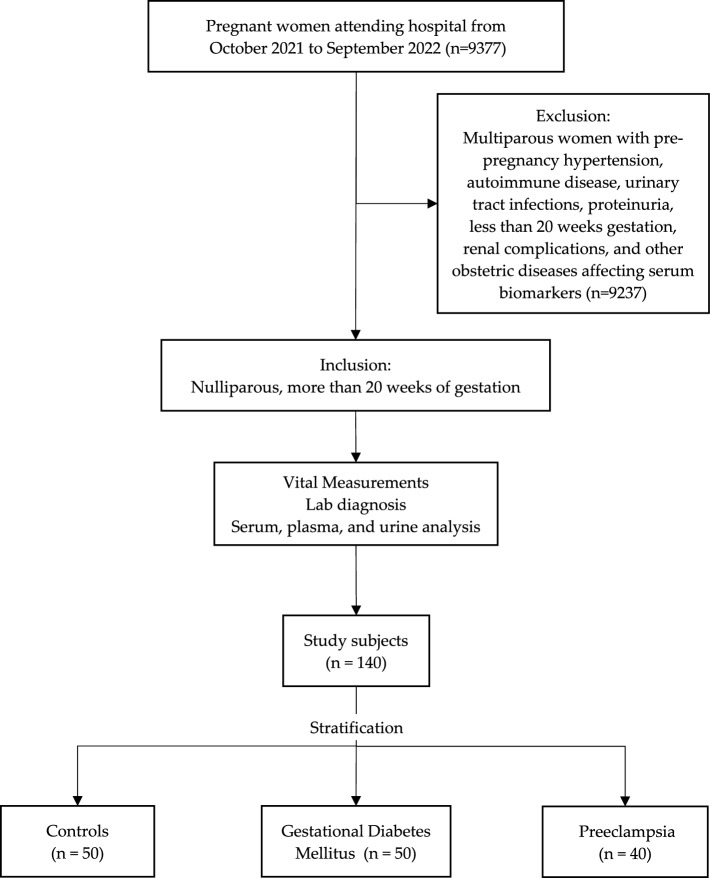
Flow chart representing study subjects recruitment and categorization.

Nulliparous women arriving for antenatal service at AlKhamis Maternity and Children Hospital were consecutively screened and enrolled. The study excluded multiparous women with pre-pregnancy hypertension, autoimmune disease, urinary tract infections, proteinuria before 20 weeks of pregnancy, renal complications, and other obstetric diseases affecting serum biomarkers. A total of 140 pregnant women with not less than 20 weeks of pregnancy were included in the study from October 2021 to September 2022. Based on clinical and laboratory diagnosis, the total studied subjects were stratified into GDM (n = 50), PE (n = 40), and controls (n = 50) with no complications reported (Fig. 1). According to IADPSG recommendations^3^, a 2-h 75-g oral glucose tolerance test (OGTT) was performed to screen GDM in the study subjects. GDM is diagnosed when fasting plasma glucose = 92 mg/dl, 1-h OGTT = 180 mg/dl, 2-h OGTT ≥ 153 mg/dl^3^. According to the American Diabetes Association ^16^, PE is characterized by > 140/90 mmHg systolic and diastolic blood pressure on more than two occasions after 20 weeks of gestation with the addition of > 0.3gm of protein in 24 h of urine test. The recent readings of systolic and diastolic blood pressure were recorded at least 4 h apart. BMI was calculated using the metric formula weight in kilograms divided by height in meters squared.

### Sample collection and analysis

Blood samples were collected by venipuncture in different color-coded labeled vacutainers. To estimate glucose levels, 3 mL of blood was collected in a purple-colored vacutainer coated with EDTA to isolate the plasma. For the biochemical analysis of TC, Tg, HDL-C, and LDL-C, a red-colored vacutainer was used to collect blood that was then centrifuged at 3000 rpm for 10 min in a tabletop centrifuge to separate the serum. The obtained serum was analyzed using an automatic analyzer, according to manufacturer instructions (Merck Ltd.). 24-h urine samples were received in wide-mouth, clean plastic universal containers for analysis. The proteinuria-positive cases identified using the dipstick method were further analyzed for urinary albumin and creatinine by Roche analyzer to estimate urinary albumin creatinine ratio (uACR). All laboratory results with vital signs were recorded.

### Statistical analysis

Statistical analysis was performed using SPSS version 25. Data of studied biochemical parameters were presented as Mean ± SD (standard deviation) values. All the groups were compared using one-way ANOVA at 0.0001 & 0.05 significance level, followed by Post hoc Tukey’s honest test and indicated by superscript at 0.001 and 0.05 significance level. Multiple regression analysis was implemented to assess the association among study variables. Further, in Model 1, the association of 2 h OGTT with blood pressure (systolic and diastolic) and lipid profile parameters was done collectively. In Model 2, the associations were performed with individual variables excluding the irrelevant variables of Model 1. Both models were presented as coefficients, standard errors, and confidence intervals.

## Results

The results of the present study include data analysis of 140 study subjects. According to physician diagnosis, subjects were grouped into controls (n = 50), GDM (n = 50) and PE (n = 40). Biochemical parameters were analyzed and presented as Mean ± SD values in Table 1.

**Table 1:** Mean ± SD values of studied parameters and their comparison among different groups.

	CONTROL (n = 50)	GDM (n = 50)	PE (n = 40)	*p* value
Maternal age (years)	33 ± 4^a^	38.74 ± 4.3^b^	40.8 ± 3.12^b^	< 0.0001
Gestational age (weeks)	26 ± 1^a^	26.88 ± 1.66^b^	27.28 ± 1.18^b^	< 0.01
Vitals				
Heart rate beats/min	88.8 ± 6.27^a^	104.4 ± 2.59^b^	113.4 ± 1.08^c^	< 0.0001
Respiratory rate breaths/min	19.2 ± 1.5^a^	20.7 ± 1.11^b^	24.3 ± 1.03^c^	< 0.0001
SpO_2_	96.6 ± 1.83^a^	94.8 ± 0.6^b^	92.5 ± 1.15^c^	< 0.0001
Temperature ^◦^C	34.9 ± 0.6^a^	34.9 ± 0.8^a^	37.4 ± 0.4^b^	< 0.0001
BMI	27.28 ± 1.13^a^	32.32 ± 2.37^b^	34.15 ± 2.73^b^	< 0.0001
Systolic mm/Hg	120.9 ± 3.3^a^	137.6 ± 1.67^b^	161 ± 3.2^c^	< 0.0001
Diastolic mm/Hg	81.2 ± 1.77^a^	103.26 ± 6.15^b^	106.6 ± 2.19^c^	< 0.0001
Laboratory analysis				
FPG mg/dl	89.32 ± 2.4^a^	101.73 ± 5.51^b^	131.74 ± 5.07^c^	< 0.0001
1 h OGTT mg/dl	110.3 ± 7.7^a^	190 ± 12.45^b^	180.75 ± 14.92^c^	< 0.0001
2 h OGTT mg/dl	120.6 ± 2.9^a^	163.24 ± 4.66^b^	152.7 ± 2.15^c^	< 0.0001
Urinary albumin mg/dl	2.15 ± 1.21^a^	2.94 ± 2.22^b^	334.25 ± 33.27^c^	< 0.0001
Urinary creatinine mmol/l	2.02 ± 0.32^a^	1.72 ± 0.45^b^	10.43 ± 0.81^c^	< 0.0001
uACR mg/mmol	1.08 ± 0.6^a^	8.3 ± 3.2^b^	32.13 ± 2.11^c^	< 0.0001
TC mg/dl	130.7 ± 2.9^a^	143.2 ± 4.19^b^	142.88 ± 2.33^b^	< 0.0001
Tg mg/dl	58.6 ± 3.09^a^	73.34 ± 4.69^b^	78.03 ± 4.83^c^	< 0.0001
HDL-C mg/dl	28.6 ± 3.2^a^	20.14 ± 3.18^b^	21.6 ± 1.41^c^	< 0.0001
LDL-C mg/dl	76.96 ± 3.23^a^	90.82 ± 3.02^b^	96.45 ± 2.58^c^	< 0.0001
VLDL-C mg/dl	11.73 ± 0.62^a^	14.66 ± 0.94^b^	15.61 ± 0.97^c^	< 0.0001

Mean ± SD values not sharing same superscripts differ significantly at 0.0001.

*GDM* Gestational diabetes mellitus, *PE* Preeclampsia, *BMI* Body mass index, *FPG* Fasting plasma glucose, *OGTT* Oral glucose tolerance test, *uACR* Urinary albumin creatinine ratio, *TC* Total cholesterol, *Tg* Triglycerides, *HDL-C* High density lipoprotein cholesterol, *LDL-C* Low-density lipoprotein cholesterol, *VLDL-C* Very low-density lipoprotein cholesterol.

Mean ± SD values of all the groups were compared using ANOVA and found to be significantly different from each other at *p* < 0.01 and *p* < 0.001. Further, the post hoc test allocates the difference among groups and is represented with alphabetical superscripts. Mean ± SD values not sharing the same superscript differ significantly at 0.001. Recorded vitals were significantly different among all groups except the body temperature of controls and GDM groups. Maternal age and gestational age showed no difference in GDM and PE groups. Increased BMI was observed in the GDM and PE groups with no difference. The systolic and diastolic blood pressure was observed to be elevated in the PE group and differed significantly from the controls and GDM group, *p* < 0.0001. The 2 h OGTT-glucose and uACR were relatively high in GDM and PE groups respectively, which differ from other groups. In the lipid index, the mean of TC was higher in GDM and PE with no difference, while Tg, LDL-C and VLDL-C markedly increased in PE group and HDL-C in controls compared to others.

This study is based on the hypothesis that maternal glucose levels are associated with alteration in blood pressure and lipid profile. To test this hypothesis, regression analysis was performed. Table 2 depicts 85% variance in blood pressure F(2,47 = 139.8, *p* < 0.001) and lipid indices F(5,45 = 87.22, *p* < 0.001) of the GDM group. In the PE group, 71% of variance on blood pressure F(2,37 = 48.8, *p* < 0.001) and 33% of the variance in lipid profile F(5,35 = 6.4, *p* < 0.001) can be accounted by GDM.

**Table 2:** Adjusted R^2^ values of 2-h OGTT on overall blood pressure and lipid profile determinants among studied groups.

	CONTROL (n = 50)	GDM (n = 50)	PE (n = 40)
Blood pressure (systolic & diastolic)	0.02	0.85*	0.71*
Lipid profile determinants (TC, Tg, HDL-C, LDL-C, VLDL-C)	− 0.10	0.85*	0.33*

*GDM* Gestational diabetes mellitus, *PE* Preeclampsia, *OGTT* Oral glucose tolerance test, *TC* Total cholesterol, *Tg* Triglycerides, *HDL-C* High density lipoprotein cholesterol, *LDL-C* Low-density lipoprotein cholesterol, *VLDL-C* Very low-density lipoprotein cholesterol.

*p value < 0.001.

Looking at Table 3 where glucose levels (2 h OGTT) reveal unique contribution on individual parameter, the result shows the regression coefficients of two models in control; GDM and PE group.

**Table 3:** Multiple regression models of 2-h OGTT on blood pressure and lipid profile determinants among studied groups.

	CONTROL (n = 50)		GDM (n = 50)		PE (n = 40)	
	Model 1	Model 2	Model 1	Model 2	Model 1	Model 2
Systolic mm/Hg	− 0.03 (0.12) − *0.2 to 0.1*	− 0.08 (0.12) − *0.3 to 0.1*	2.39 (0.21)*** *1.9 to 2.8*	2.57 (0.15)*** *2.2 to 2.8*	0.55 (0.06)*** *0.4 to 0.6*	0.57 (0.05)*** *0.4 to 0.6*
Diastolic mm/Hg	0.37 (0.23) − *0.1 to 0.8*	0.38 (0.22) − *0.07 to0.8*	0.07 (0.05) − *0.04 to0.1*	0.51 (0.07)*** *0.3 to 0.6*	0.07 (0.08) − *0.1 to 0.2*	0.31 (0.15)** *0.01 to 0.6*
TC mg/dl	0.01 (0.14) − *0.2 to 0.3*	0.009 (0.14) − *0.2 to 0.2*	0.37 (0.11)*** *0.1 to 0.6*	0.98 (0.07)*** *0.8 to 1.1*	0.015 (0.13) − *0.2 to 0.2*	0.25 (0.14) *0.0 to 0.5*
Tg mg/dl	− 0.04 (0.14) − *0.3 to 0.2*	− 0.05 (0.13) − *0.3 to 0.2*	0.23 (0.12) − *0.01 to 0.4*	0.88 (0.06)*** *0.7 to 1*	0.19 (0.07)** *0.04 to 0.3*	0.26 (0.05)*** *0.1 to 0.3*
HDL-C mg/dl	0.03 (0.13) − *0.2 to 0.3*	0.01 (0.12) − *0.2 to 0.2*	− 0.25 (0.14) − *0.5 to 0.03*	− 1.2 (0.11)*** − *1.4 to* − *1*	0.43 (0.21)* *0.0 to 0.8*	0.73 (0.21)*** *0.2 to 1.1*
LDL-C mg/dl	0.07 (0.13) − *0.1 to 0.3*	0.07 (0.12) − *0.1 to 0.3*	0.38 (0.18)* − *0.0 to 0.7*	1.37 (0.09)*** *1.1 to 1.5*	0.06 (0.12) − *0.1 to 0.3*	0.31 (0.12)* *0.0 to 0.5*
VLDL-C mg/dl		− 0.25 (0.67) − *1.6 to 1.1*		4.4 (0.3)*** *3.7 to 5*		1.31 (0.29)*** *0.7 to 1.9*

Numeric values are regression coefficients followed by robust standard errors in parenthesis and a 95% confidence interval (lower to upper range) in italics.

*TC* Total cholesterol, *Tg* Triglycerides, *HDL-C* High density lipoprotein cholesterol, *LDL-C* Low-density lipoprotein cholesterol, *VLDL-C* Very low-density lipoprotein cholesterol.

***p < 0.0001; **p < 0.01; *p < 0.05.

An insignificant effect of 2 h-OGTT-glucose was seen on the controls' blood pressure and lipid profile. In the GDM group, a significant positive effect of 2 h-OGTT was observed on systolic blood pressure (model 1: t value = 11.2; model 2: t value = 16.5), diastolic blood pressure (model 2: t value = 6.54) and lipid profile determinants (TC- model 1 & 2: t value = 3.25 & 13.4 respectively; Tg- model 2: t value = 13.7; HDL-C- model 2: t value =  − 11.0; LDL-C- model 1 & 2 t-value = 2.0 &13.7 respectively; VLDL-C: t value = 13.7); except HDL-C showing a negative impact at *p* < 0.001. In the PE group, 2 h-OGTT-glucose portrays a significant effect on systolic blood pressure (model 1: t value = 9.13; model 2: t value = 9.89), diastolic blood pressure (model 2: t value = 2.12), Tg (model 1 & 2: t value = 2.6 & 4.4 respectively) , HDL-C (model 1 & 2: t value = 1.9 & 3.4 respectively), LDL-C (model 2: t value = 2.5), and VLDL-C (model 2: t value = 4.4), at *p* < 0.001, *p* < 0.01, and *p* < 0.05.

## Discussion

GDM demonstrates imbalanced vascular, metabolic, and inflammatory processes by distinguishably accelerated concentrations of inflammatory molecules and placental genes encoding for inflammatory mediators^17,18^. GDM is a known risk factor for stillbirth, fetal macrosomia, fetal structural anomalies, premature delivery, and gestational hypertensive disorders^19^. GDM is often diagnosed in the second trimester of pregnancy. Over time, excessive glucose levels may be attributed to gestational hypertension disorders and related intricacies^20^.

In the present study, advanced maternal age is observed in the GDM and PE groups, consistent with the studies suggesting that older age can be a risk factor for the onset of GDM^21,22^. Other risk factors like multiple pregnancies and higher pre-pregnancy BMI are also disclosed in the pathogenesis of PE in women with early-onset GDM^23^. Alfadhli et al. reported the high prevalence of GDM in Saudi women due to older age, increased BMI, and hypertension^24^. A cohort study assessing the risk of PE in women diagnosed with GDM documented that the risk was eight times higher when GDM is detected during 20 weeks of pregnancy^25^. Our study participants in both GDM and PE groups have 26–28 weeks of pregnancy and elevated BMI with no statistical difference, suggesting that GDM in later weeks of pregnancy is likely to worsen the condition. Progressive maternal and gestation age altogether may be the reason for GDM and its exaggerated manifestation like PE. A study by Erkamp et al.^20^ reported the positive correlation of non-fasting glucose concentrations with blood pressure in early pregnancy, but not in later pregnancy.

Diabetes and hypertension frequently coexist and share risk factors and disease etiologies including genetics, obesity, insulin resistance, and inflammation^26^. The current study observed a significant impact of OGTT glucose on systolic and diastolic blood pressure in GDM and PE women. However, significant differences in OGTT glucose and systolic and diastolic blood pressure in GDM and PE groups suggest that the probable reason could be persistently elevated glucose to later trimester affecting the blood pressure. Various studies expressed that the risk of gestational hypertensive disorders and PE is significantly raised by GDM^6,7^. An earlier prospective research of healthy nulliparous women revealed that the probability of PE was positively linked with fasting blood glucose concentrations, even within the normal range^27^. On the contrary, a study described that normal glucose concentrations in early pregnancy are not associated with gestational hypertensive disorders^20^. It has been suggested that moderate exercising in pregnant women with GDM helps in controlling pregnancy weight and blood sugar levels, but it has little impact on the development of PE^28,29^. In a recent randomized trial, it was observed that gestational diabetics receiving proper immediate treatment before 20 weeks had a lower incidence of adverse effects on neonates^30^.

The presence of persistently increased glucose concentrations during pregnancy have a dramatic influence on lipid profile^1^. In the present line of work, GDM and PE groups present that hyperlipidemic profile is significantly affected by OGTT glucose (*p* < 0.001). Numerous effects like maternal risk of chronic hypertension, cardiovascular disease, diabetes, perinatal adverse events, and increased offspring BMI are noticed upon GDM complicated by PE^31,32^. Excessive lipid accumulation inside endothelial cells can lead to endothelial dysfunction and reduced prostacyclin release, which is a crucial factor in the development of PE^33,34^. However, the effect of GDM on dyslipidemia has been disregarded in routine screening, which might be the risk factor for PE. Dyslipidemia is a metabolic disorder defined by elevated levels of LDL-C, Tg, and low levels of HDL-C^1^. Our regression models in the GDM and PE groups align with other research reports which have documented an increase in TC, Tg, LDL-C, and a decrease in HDL-C concentrations associated with PE risk^35–37^. The National Health and Nutrition Examination Survey's research of parous women revealed no significant correlation between GDM and rising levels of LDL-C^38^. On the other hand, women who have had GDM exhibit a greater frequency of dyslipidemia than their normoglycemic counterparts^39^. Conflicting results have been reported while elucidating the relation between maternal lipid profile and PE^40–43^. Recent evidence reported deregulations of feto-maternal lipid metabolism are related to the pathogenesis of PE. Wojcik-Baszko et al.^44^ found dyslipidemia positively associated with PE^11^.

Nulliparous subjects diagnosed with gestational diabetes after 20 weeks of pregnancy strengthen the present study by depicting the need for monitoring blood glucose levels in early pregnancy to prevent the risk of developing maternal and fetal complications. It is important to note that this study represents its regional importance and has not been previously documented in Abha. However, the study is monocentric, and risk factors such as early pregnancy glucose concentrations and gestational weight gain were not assessed, which may affect the generalizability of the results. Examining these factors could provide valuable insights into the links between blood pressure, disrupted lipid levels, and other hypertensive disorders of pregnancy.

## Conclusions

The current study indicates that glucose intolerance during the later weeks of pregnancy is associated with gestational hypertension and hyperlipidemia as a risk factor for PE. Further research is needed for a detailed assessment of maternal glucose metabolism at various pregnancy stages including the use of more sensitive markers such as C-peptide and their relation to pregnancy-related hypertensive disorders.

## Data Availability

All data is included in this article.

## Abbreviations

GDM: Gestational diabetes mellitus
PE: Preeclampsia
OGTT: Oral glucose tolerance test
TC: Total cholesterol
Tg: Triglyceride
HDL-C: High density lipoprotein-cholesterol
LDL-C: Low density lipoprotein-cholesterol
uACR: Urinary albumin creatinine ratio
IADPSG: International Association of Diabetes and Pregnancy Study Groups
HAPO: Hyperglycemia and Adverse Pregnancy Outcome
